# Safety of Nintedanib in a Patient with Chronic Pulmonary Fibrosis and Kidney Disease

**DOI:** 10.3390/ph17091147

**Published:** 2024-08-30

**Authors:** Marta Maggisano, Lucrezia Mondini, Maria Chernovsky, Paola Confalonieri, Francesco Salton, Nicolò Reccardini, Metka Kodric, Pietro Geri, Marco Confalonieri, Michael Hughes, Rossella Cifaldi, Barbara Ruaro

**Affiliations:** 1 Pulmonology Unit, Department of Medical Surgical and Health Sciences, University of Trieste, Hospital of Cattinara, 34149 Trieste, Italy; 2 Division of Musculoskeletal and Dermatological Sciences, Faculty of Biology, Medicine and Health, The University of Manchester Salford Royal NHS Foundation Trust, Manchester M6 8HD, UK

**Keywords:** nintedanib, safety, pulmonary fibrosis, chronic kidney disease

## Abstract

Nintedanib, an intracellular inhibitor that targets multiple tyrosine kinase, is an important drug for the treatment of pulmonary fibrosis. Until now, no studies have been published reporting the nintedanib tolerability or its efficacy in patients with chronic pulmonary lung disease and chronic kidney disease comorbidity. The safety, efficacy and pharmacokinetics of nintedanib have not been studied in patients with severe renal impairment (creatinine clearance < 30 mL/min) and for this reason it is contraindicated in these patients. We describe a case of use of nintedanib in a patient affected by idiopathic pulmonary fibrosis (IPF) who started, from 2022, nintedanib 150 mg twice a day with careful monitoring of liver and kidney function. Due to the onset of stage 3/4 chronic kidney disease associated with proteinuria, nintedanib was suspended for two months, and the patient received Prednisone at a dose of 12.5 mg/day. During the two months of suspension, the renal function did not improve, unlike the respiratory status worsened. In the past a renal biopsy was performed which showed no correlation with nintedanib use. Nintedanib therapy started again following the decline in lung function and desaturation below 90% in the 6-min walking test (6MWT). Patient showed a good tolerability of nintedanib with sporadic episode of diarrhea and an improvement of pulmonary function leading to a stable state of chronic pulmonary fibrosis disease. For this reason, in mutual agreement with the patient, we decided to maintain nintedanib therapy even when the patient required hemodialysis. No toxic effects appeared. This case report revealed the safety of nintedinab in patient with concomitant kidney failure, but more studies are necessary.

## 1. Introduction

Idiopathic Pulmonary Fibrosis (IPF) is a rare disease affecting the lungs, characterised by progressive, non-reversible lung fibrosis, which typically affects patients over the age of 60, predominantly of the male sex, with incidence increasing with each year. IPF is a highly debilitating disease, with devastating repercussions on the patients’ physical and mental well-being ranging from cognitive decline due to prolonged hypoxia to social isolation and anxiety when confronted with the diagnosis. The median survival rate varies from two to three years after diagnosis and is related to the progression rate, which may or may not have intermittent periods of clinical and functional stability [1]. The clinical manifestations and progression of IPF are oftentimes nonspecific and are very variable, although some symptoms such as persistent dry coughing and worsening dyspnoea are found in near all patients, especially in later stages of progression [1,2]. The neurological and mental health manifestations of IPF are significant and multifaceted, reflecting the complex interplay between respiratory function and overall well-being. While in most patients’ with IPF the disease progression is a slow and steady process of clinical, functional, and radiological deterioration, few patients experience acute exacerbations of IPF (AE-IPF), which are the cause of roughly half of the IPF related deaths.

Up to now, no target therapy able to eradicate the cause of IPF exists. In 2014, the so called antifibrotic drugs, pirfenidone and nintedanib, has been introduced and approved for use in IPF. Both drugs have been approved by the Food and Drug Administration (FDA) for IPF treatment, as numerous clinical trials such as INPULSIS, ASCEND, CAPACITY and also real-life studies have shown that they can slow the progression of the disease, particularly by reducing the decline in lung function and the frequency of acute exacerbations, thereby also improving patients’ quality of life [3,4,5]. Despite anti-fibrotic therapy, IPF disease remains progressive, and ultimately results in death in many cases. A recent meta-analysis revealed that anti-fibrotic therapy increases the median survival length after diagnosis, offering protection against the rate of decline in forced vital capacity (FVC) in progressive lung fibrosis with no differences between the two anti-fibrotic agents currently in clinical use [4]. Tyrosine kinase inhibitor (TKI) drugs are a group of small molecules which share a common action mechanism, but differ in target receptors. In the case of Nintedanib, inhibition of the fibroblast growth factor is responsible for the haematological side effects of most of TKIs like anemia, thrombopenia and neutropenia, the most common extraheamatologic adverse effects are edema, nausea, hypothyroidism, vomiting and diarrhoea. It is a relatively safe drug with most side effects being mild and manageable with symptomatic therapy such as loperamide in case of diarrhea. It is important to note that while increased risk of bleeding (especially for patients undergoing simultaneous oral anticoagulant therapy) has been hypothesized for Nintedanib, it has not been proven in clinical trial [5,6].

Nintedanib is a small molecule able to bind to tyrosine-kinase receptors such as platelet-derived growth factor receptor (PDGFR), fibroblast growth factor receptors (FGFR), vascular endothelial growth factor receptor (VEGFR), and Src family kinase [7]. Tyrosine kinases are critical regulators of various cellular processes, including proliferation, differentiation, and apoptosis. In the context of fibrosis, they play a pivotal role in the signaling pathways that mediate the activation and proliferation of fibroblasts, which are key effector cells in the fibrotic process. In IPF, lung fibroblasts undergo activation and transdifferentiation into myofibroblasts, the cells responsible for excessive collagen deposition and extracellular matrix remodeling. Tyrosine kinases, such as those belonging to the receptor tyrosine kinase (RTK) family, can promote this activation by transducing signals from various growth factors (e.g., platelet-derived growth factor [PDGF], fibroblast growth factor [FGF]) that are overexpressed or dysregulated in IPF. Tyrosine kinases are also involved in the regulation of inflammatory responses. Inflammation can contribute to the development and progression of fibrosis. Certain kinases facilitate the recruitment and activation of inflammatory cells, which can further exacerbate lung injury and promote fibrosis. The Wnt signaling pathway, which is influenced by tyrosine kinases, has been implicated in the pathogenesis of IPF. Activation of this pathway in lung fibroblasts can lead to enhanced fibrosis. Specifically, signaling through Wnt can promote myofibroblast differentiation and increase collagen synthesis. In IPF, there may be dysregulation or aberrant activation of tyrosine kinases leading to an imbalance in profibrotic and antifibrotic signals. Understanding these aberrations may provide insights into targeted therapies that could halt or reverse the fibrotic process. Given the role of tyrosine kinases in divergent aspects of fibrosis, these kinases represent potential therapeutic targets. Some tyrosine kinase inhibitors (TKIs) have shown promise in preclinical and clinical studies for treating IPF by reducing fibroblast proliferation, collage deposition, and overall lung fibrosis. The role of tyrosine kinases in idiopathic pulmonary fibrosis is multifaceted, encompassing cellular signaling, fibroblast activation, and the inflammatory response. As research in this area continues, targeting tyrosine kinase pathways may offer new therapeutic avenues for managing IPF and improving patient outcomes. Further studies are needed to elucidate the specific kinases involved, their mechanisms of action, and how they can be effectively targeted in the context of idiopathic pulmonary fibrosis.

**Figure 1:**
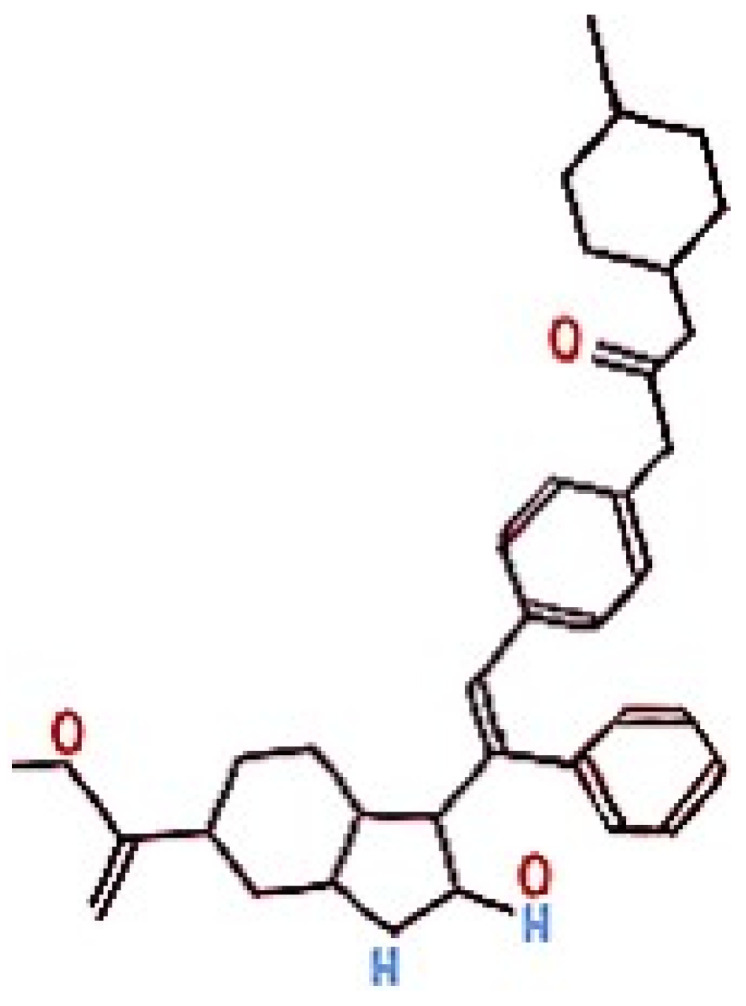
Molecular structure of Nintedanib. The red O represents exposed oxygen atoms, the grey H represents hydrogen atoms (revised by Chernovsky M.).

Nintedanib is a small molecule drug with the chemical formula C21H24N4O4S. It is used primarily in the treatment of idiopathic pulmonary fibrosis and certain types of cancer. The structure of Nintedanib includes a complex arrangement of rings and functional groups and is an ethylsulfonate salt, shown in Figure 1 [8].

Small molecules such as nintedanib are lypophilic in nature and are unionized in neutral pH and are capable of nonspecific binding to human liver microsomes [9]. This might pose a problem for patients with a terminal kidney disease especially in patients undergoing dialysis, as it affects not only the clearance of drugs eliminated via urine but shifts the metabolism of drugs eliminated via the hepato-biliary system because of increased uraemia levels on both protein binding, drug transporting canal activity and hepatic enzyme activity [10]. In addition, a case report indicating the development of anti-Basal Membrane (BM) antibodies causing acute kidney injury by binding to the basal membrane of the glomeruli, disrupting its integrity and function and leading to glomerulonephritis and acute kidney injury, which, in particularly severe cases or untreated cases, may lead to severe kidney failure, raising awareness to the possibility of autoimmune acute kidney injury which would be especially devastating to patients with an already impaired renal function [11,12]. To the best of our knowledge, one report of a patient undergoing nintedanib treatment with chronic kidney disease has been published so far, though the patient was not dialysis dependent as their estimated Glomerular Filtration Rate (eGFR, according to the Cockroft-Gault equation of creatinine clearance) were higher than 15 mL/min [13].

Here we describe a case-study on nintedanib tolerability and efficacy in a patient with chronic pulmonary lung disease and chronic kidney disease, on dialysis therapy, displaying the autoimmune thyroid diseases (AITD) and type-2 diabetes comorbidities.

## 2. Detailed Case Description

We describe a 77-year-old nonsmoking male patient with a medical history of hypertension, type 2 diabetes mellitus, prostatic hypertrophy, autoimmune thyroiditis, congestive heart failure and stage 5 chronic renal failure. All baseline data are shown in Table 1.

**Figure 2:**
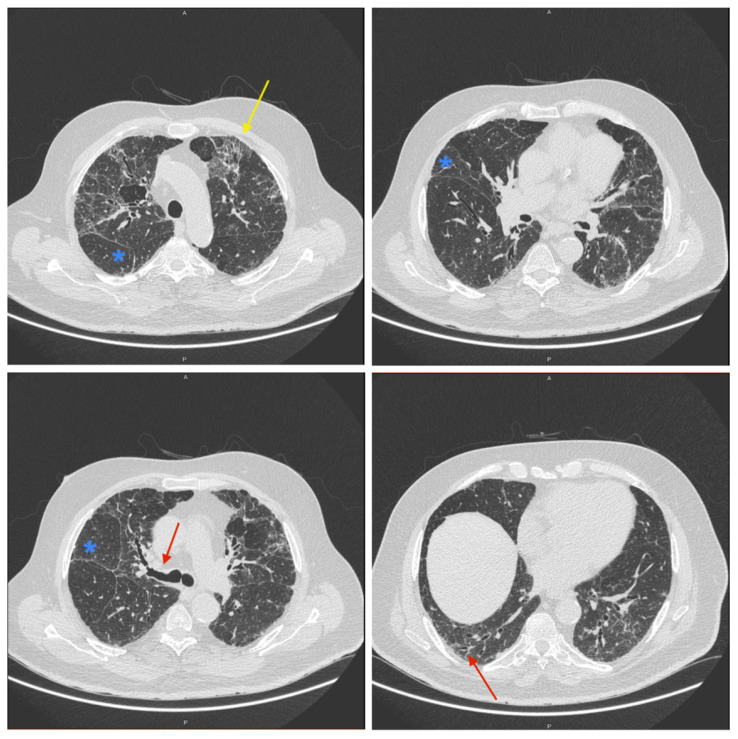
Chest high-resolution computed tomography (HRCT) performed at T0 showing traction bronchiectasis (red arrow), ground glass opacities (yellow arrow) and septal thickening (blue asterisk).

In 2014, he underwent right hemicolectomy surgery for right colon adenocarcinoma, which was then followed by adjuvant chemotherapy with oxaliplatin and capecitabine for two months. During oncological follow-up, the patient underwent a CT scan of the abdomen, which showed discrete signs of interstitial disease, so a subsequent high-resolution computed tomography (HRCT) scan of the chest was performed showing discrete signs of interstitiopathy with reticular appearance due to thickening of inter- and intralobular septa to which bronchiectasis with slightly thickened bronchial walls and lobular air trapping. HRCT results, shown in Figure 2, corresponded to radiological features of probable usual interstitial pneumonia (UIP), the hallmark of IPF, as described in detail in the 2018 guidelines for diagnosis of IPF [14] and in the 2022 update on IPF diagnosis [15]. The patient had already ultimate chemotherapy cycle since three years, so as some cases of pulmonary fibrosis, also with UIP pattern, associated with Oxaliplatin intake are well described in literature [16], we decided to monitor the radiological picture over time. Despite discontinuation of the drug, which had led to the hypothesis of secondary pulmonary fibrosis, there was radiological progression and functional worsening, consequently drug strategies for treatment of IPF were evaluated. Despite the clinical recommendations, the patient did not agree to start the therapy with antifribotic drugs and underwent pneumological follow-up. In 2019 the patient was admitted to the Pulmonology Unit in Trieste with dyspnea on exertion, cough and a moderate-grade restrictive ventilatory deficit with severe reduction of alveolus capillary diffusion of carbon monoxide (DLCO) on previous respiratory function tests. During 2019, the patient underwent respiratory function tests that revealed moderate-grade restrictive ventilatory deficit (TLC 57%, RV 45%, FEV1/FVC 86–113%, FVC 59%, FEV1 67%, FEF 25–75% 130%) with moderate reduction in alveolar capillary diffusion of carbon monoxide (DLCO 44%). The patient performed the 6-min walk test (6MWT), which showed significant exertional desaturation (initial SpO2 94% and final 84%). He performed a bronchoscopy with bronco-alveolar-lavage (BAL) that showed the presence of lymphocytic alveolar inflammation for which Prednisone was administered at a dosage of 25 mg in combination with lansoprazole 30 mg for secondary prevention of gastric or duodenal ulcers associated with long-term non-steroidal anti-inflammatory drug (NSAID) therapy. During pulmonology follow-up, blood tests and urine analysis were performed revealing worsening of creatinine values (2 mg/dL) and proteinuria that reached the value of 3 g/24 h. Due to the nephrotic proteinuria and chronic renal failure, a renal biopsy was therefore performed, showing nephropathy with multifactorial genesis, diabetic and hypertensive, associated with focal centers of subacute tubulo-interstitial nephritis. In 2021, the patient was admitted to the pulmonology department for acute and chronic respiratory failure in exacerbation of congestive heart failure, for which oxygen therapy (2 L/min) was prescribed. At the same time, routine blood tests showed a further worsening of renal function with a creatinine of 2.36 mg/dL and an eGFR of 18 mL/min 1.73 m^2^ bringing chronic renal failure to stage 3B. In 2022, the patient had a paucisymptomatic SARS-CoV-2 infection that last for one week. The follow-up chest CT scan showed thickening of the intralobular septa and shaded areas of ground-glass-opacity, traction bronchiectasis some cystic formations in a picture referable to fibrosing pathology. Due to the radiological and clinical patterns, the case was presented in the context of multidisciplinary discussion after which it was decided to perform a fiberoptic bronchoscopy with bronchoalveolar lavage, lung biopsy and TransBronchial Needle Aspiration (TBNA) in lymphonode 11 L. Histological investigation confirmed the diagnostic suspicion of IPF, showing dense fibrosis with architectural distortion. Microscopic and culture examination was negative for mycobacteria, viruses and Aspergillus galactomannan. Due to the examinations performed and the clinical-laboratory state, at the end of June 2022 therapy with nintedanib at the dosage of 150 mg 1 cp twice a day was recommended and well tolerated by the patient. The rationale for the choice of antifibrotic drug, as usual, was shared with the patient by evaluating both his clinical situation and his preferences. The patient’s personal preferences regarding treatment, including fears regarding side effects and the desire to avoid certain types of side effects, are always taken into account. In particular, in our case study the patient preferred nintedanib both for the therapeutic regimen and the profile of possible adverse reactions of pirfenidone. In fact, nintedanib is administered at a fixed dosage of 150 mg twice a day, while pirfenidone requires an incremental dosage (up to 801 mg per day), which was complicated for the studied patient. Furthermore, pirfenidone is more frequently associated with skin rash and our patient was very afraid of this adverse event because he lives near the sea and has gardening as a hobby. After the introduction of nintedanib the prednisone has been tapering to 6.25 mg. The 6MWT improved with less final desaturation for the same distance walked (initial SpO2 96% and final 92%). Routine blood tests highlight worsening of renal function (chronic renal failure stage 4) with creatinine values of 3.21 mg/dL referred to the diuretic effect and thus to pre-renal condition. An abdominal ultrasound was performed showing chronic nephropathy without hydrourethronephrosis with multiple cystic formations. In 2023 the patient reported clinical stability with onset of productive cough, especially in the morning, with greenish color sputum sample and reduced sputum expectoration due to reduced exercise tolerance and dyspnea. Chest CT scan showed at basal level the appearance of some thickening patches, partly with consolidative appearance partly with ground glass appearance; persistence of irregular thickening of inter- and intralobular septa and traction bronchiectasis. The respiratory function tests revealed worsening of ventilation with severe grade restrictive ventilatory deficit associated with a severe reduction in alveolar capillary diffusion of carbon monoxide. At discharge the patient clinically improved, with no longer dyspnea or coughing. The patient has been taking nintedanib therapy for eight months. Due to the possibility of the chronic renal failure at stage 4/5 in association with nintedanib, it was decided to stop therapy with nintedanib for two months and continue therapy with Prednisone increasing the dose to 12.5 mg/die. After one month, the patient referred worsening of the clinical status with cough, dyspnea on mild exertion. The respiratory function tests were stable but the 6MWT was worsening with a significant oxygen desaturation of 86% without oxygen supplementation; arterial blood gases analysis highlights increased hypoxemia compared with discharged (pH 7.39, PaO_2_ 73 mmHg, PaCO_2_ 38 mmHg, HCO_3_^−^ 23, SpO_2_ 95.7%). The patient, due to the significant oxygen desaturation, decided to independently take therapy with nintedanib 150 mg twice daily after two months of stopping, a dose that was well tolerated and helped the patients to get a remission of dyspnea and coughing. The patient reinitiated therapy for fifteen months until now. Creatinine values were stable (3.42 mg/dL). After two months, the patient presented acute on chronic respiratory failure with worsening of renal failure for which the patient underwent Central Venous Catheter (CVC) insertion for initiation of hemodialysis treatment. The patient underwent fiberoptic bronchoscopy with bronchoalveolar lavage, with showed FilmArray Pneumonia Panel positive for Legionella Pneumophila for which he started targeted antibiotic therapy. Maintenance of hemodialysis treatment three times a week was planned. Respiratory symptoms improved so the patient was discharged. At the last pneumology follow-up, the patient reported stability of exertional dyspnea, modest cough with yellowish-white sputum, no fever, no wheezing, no paroxysmal nocturnal dyspnea and an improvement in renal function. Respiratory function tests were slightly improving. He is currently taking regular therapy with nintedanib 150 mg 1 cp twice a day, which it is well tolerated, oxygen therapy 1 L/min at rest and 2–3 L/min under exertion. He is also performing regularly respiratory physiotherapy with benefit.

## 3. Discussion

IPF patients are often elderly males with multiple comorbidities [17,18,19,20,21,22,23]. The impact of pulmonary and extrapulmonary comorbidities on the prognosis of IPF has been widely assessed [18]. A significant influence on quality of life and functional impairment was found with an elevated risk of death in patients with IPF [19]. Against such a background, in IPF, the chronic kidney disease (CKD) has recently been drawing attention. A recent study underlines the link between kidney and lung, the so-called kidney-lung axis, even in the early stages of kidney disease, suggesting an important role of endothelial dysfunction in the development of lung disease [20]. In fact, CKD patients have lower pulmonary function, respiratory and peripheral muscle strength values when compared with healthy individuals, which reflects negatively in the quality of life [21]. The co-existence of renal and pulmonary disease highlights the need to better define valid strategies for the management of patients with kidney and lung disease, and to individualize treatment decisions. Rarely, however, it has been found the crossover between a stage V kidney disease and IPF within the same patient. To the best of our knowledge, no cases of dialysis dependant IPF patients undergoing treatment with nintedanib have been reported to this date.

It is important to denote that IPF is rarely encountered in young adults, in a study conducted by Leuscher et al. less than 25% of patients diagnosed with IPF were under the age of 50, making them less likely to receive an accurate diagnosis due to IPF being widely considered an “elderly” disease. As such, it is difficult to accurately discuss the prognosis in younger patients affected by IPF and concomitant CKD, it undoubtedly poses a great challenge to physicians as nintedanib and pifenidone both are primarily excreted via urine, potentially excluding such patients from the first line and most efficient treatment currently available. In such cases, young patients have approximately the same survival rate as elderly patients in the absence of antifibrotic therapy [24,25].

The use of drugs in patients with terminal kidney disease is always a delicate matter, with drug doses requiring adjustment to compensate for reduced renal clearance [22]. This is true for both drugs eliminated via urine and through the biliary system [10,22,23,24,25,26,27]. In patients with terminal kidney disease, elevated level of blood urea, as well as impaired control of pH, contribute to impaired drug and protein binding (namely albumin), thus altering the hepatic clearance of the drug [10,22,23,24,25,26,27]. Dysregulated activation and inhibition of hepatic enzymes due to varying pH and blood urea levels responsible for drug metabolism too alters the hepatic clearance [10], nintedanib is a small, nonpolarized molecule binding to tyrosine kinase receptors, and is metabolised in the liver and as such should be affected by the reduced protein binding and altered enzyme activity. A previous case report in patient with severe chronic kidney disease (eGFR of 15–20 mL/min) revealed a minimal side effect of nintedanib. However, the authors suggest further research to better evaluate the nintedanib toxicity and efficacy in severe CKD [13]. Interestingly, in a previous report a patient undergoing nintedanib treatment for IPF has developed glomerulonephritis and anti-basal membrane antibodies within a few months after beginning treatment, resulting in acute kidney injury with a drastic increase in blood creatinine levels [11]. Before starting nintedanib, the patient had a nephrotic proteinuria and chronic renal failure for which he performed a renal biopsy that showed a diabetic and hypertensive nephropathy associated with focal centers of subacute tubulointerstitial nephritis, bereft of the typical basal membrane damage characteristic of nintedanib induced glomerulonephritis. For this reason, we considered the nephropathy already present in the patient and, therefore, independent of the use of nintedanib. Due to clinical and functional respiratory state of the patient, nintedanib was started under strict clinical, laboratory and functional monitoring and was well tolerated except for the evidence of a progression of chronic renal failure, so we decided to stop it. During the two months of suspension of nintedanib, the renal function did not improve, unlike the respiratory status worsened and the patient decided to independently resume therapy with nintedanib, obtaining an improving of dyspnea and coughing. For this reason, in mutual agreement with the patient, we decided to maintain nintedanib therapy even when the patient required hemodialysis without the appearance of toxic effects.

To our knowledge, this is the first case reporting the efficacy and safety of nintedanib therapy in an IPF patient affected by a severe kidney disease on dialysis. Our clinical case offers an important contribution to the real-life use of nintedanib, even if it refers to the description of a single patient. It is well known that case reports focusing on a limited number of individual cases cannot permit to generalize the findings to a broader population. For the safety use of nintedanib in patients with renal problems and for excluding any adverse events, larger clinical studies and data on a larger number of subjects are needed, taking into account the low incidence of this disease. A retrospective real-life, multicentre case-control study is needed to obtain more information on the use of nintedanib in patients with idiopathic pulmonary fibrosis and renal disease.

## 4. Conclusions

This case describes the safe and effective use of nintedanib in a patient with both chronic pulmonary lung disease and chronic renal failure on hemodialysis. Due to the limited options for antifibrotic therapy in IPF patients with chronic kidney disease, nintedanib treatment 150 mg twice daily could be suggested in patients with concomitant failure of lungs and kidneys undergoing hemodialysis, under strict clinical, laboratory and functional monitoring, but more studies are necessary. Little clinical data available on patients with chronic renal failure and concomitant IPF as both conditions have a median survival rate of a few years from hemodialysis treatment start and diagnosis respectively. Future treatment improvements would, hopefully, allow for a longer survival rate and quality of life for these patients.

## Figures and Tables

**Table 1:** Patient baseline characteristics.

Baseline Anamnestic Data	
Sex	M
Age	77
Smoking habits	Non-smoker
Comorbidities	Chronic renal failure St.5, DM2 ^1^, Systemic HTN ^2^, HF ^3^, BPH ^4^, autoimmune thyroiditis, AdenoK of right colon undergoing haemicolectomy and subsequent adjuvant CT ^5^
Ongoing pharmacological treatment	Bisoprolol, Enalapril, Furosemide, Ezetimibe/simvastatin, Nifedipine, Citalopram, Colecalciferol, Dutasteride, Lansoprazole, Prednisone, Sevelamer, Dulaglutide, Oxygen therapy, Nintedanib
Baseline blood exam at baseline	
Red blood cell count (RBC)	4.26 × 10^6^/μL
Hemoglobin (Hb)	12 g/dL
White blood cell count (WBC)	6.64 × 10^3^/μL
Platelets (PLT)	181 × 10^3^/μL
Creatinine	2.18 mg/dL
Sodium	134 mEq/L
Potassium	4.43 mEq/L
Urea	60 mg/dL
Baseline respiratory function	
FEV_1_/FVC	0.83
TLC ^6^	57%
FEV_1_ ^7^	67%
FVC ^8^	59%
DLCO ^9^	44%

^1^ DM2: Diabetes mellitus, ^2^ HTN: Hypertension, ^3^ HF: heart failure, ^4^ BPH: Benign prostatic hyperplasia, ^5^ CT: chemotherapy, ^6^ TLC: total lung capacity, ^7^ FEV_1_: Forced expiratory volume, ^8^ FVC: Forced vital capacity, ^9^ DLCO: diffusing capacity for carbon monoxide.

## Data Availability

Deidentified participant data will be made available upon motivated request to the Corresponding Author.
